# Topical rapamycin reduces markers of senescence and aging in human skin: an exploratory, prospective, randomized trial

**DOI:** 10.1007/s11357-019-00113-y

**Published:** 2019-11-25

**Authors:** Christina Lee Chung, Ibiyonu Lawrence, Melissa Hoffman, Dareen Elgindi, Kumar Nadhan, Manali Potnis, Annie Jin, Catlin Sershon, Rhonda Binnebose, Antonello Lorenzini, Christian Sell

**Affiliations:** 1 Department of Dermatology, Drexel University College of Medicine, Philadelphia, PA USA; 2 Department of Medicine, Drexel University College of Medicine, Philadelphia, PA USA; 3 Department of Pathology and Laboratory Medicine, Drexel University College of Medicine, 245 N 15th Street, Philadelphia, PA 19102 USA; 4 Department of Biomedical and Neuromotor Sciences, University of Bologna, Bologna, Italy; 5 Department of Biochemistry, Drexel University College of Medicine, Philadelphia, PA USA

**Keywords:** rapamycin, aging, senescence, mTOR, keratoses, photoaging

## Abstract

Aging is a major risk factor for the majority of human diseases, and the development of interventions to reduce the intrinsic rate of aging is expected to reduce the risk for age-related diseases including cardiovascular disease, cancer, and dementia. In the skin, aging manifests itself in photodamage and dermal atrophy, with underlying tissue reduction and impaired barrier function. To determine whether rapamycin, an FDA-approved drug targeting the mechanistic target of rapamycin (mTOR) complex, can reduce senescence and markers of aging in human skin, an exploratory, placebo-controlled, interventional trial was conducted in a clinical dermatology setting. Participants were greater than 40 years of age with evidence of age-related photoaging and dermal volume loss and no major morbidities. Thirty-six participants were enrolled in the study, and nineteen discontinued or were lost to follow-up. A significant (*P* = 0.008) reduction in p16^INK4A^ protein levels and an increase in collagen VII protein levels (*P* = 0.0077) were observed among participants at the end of the study. Clinical improvement in skin appearance was noted in multiple participants, and immunohistochemical analysis revealed improvement in histological appearance of skin tissue. Topical rapamycin reduced the expression of the p16^INK4A^ protein consistent with a reduction in cellular senescence. This change was accompanied by relative improvement in clinical appearance of the skin and histological markers of aging and by an increase in collagen VII, which is critical to the integrity of the basement membrane. These results indicate that rapamycin treatment is a potential anti-aging therapy with efficacy in humans.

Trial registration ClinicalTrials.gov Identifier: NCT03103893.

**Electronic supplementary material:**

The online version of this article (10.1007/s11357-019-00113-y) contains supplementary material, which is available to authorized users.

## Introduction

The potential for interventions targeting the aging process has been exploded in recent years. Preliminary clinical trials of metformin (de Kreutzenberg et al. 2015), rapamycin (Kraig et al. 2018), and nicotinamide adenine dinucleotide supplementation (Martens et al. 2018) as anti-aging interventions are based on exciting results from cognition and late-life function studies in preclinical models (Maiese 2014; Richardson et al. 2015; Wang et al. 2014). Inhibition of the mechanistic target of rapamycin (mTOR) pathway using rapamycin, an FDA-approved drug used clinically to inhibit solid organ allograft rejection, is one of the most promising anti-aging interventions. It has been demonstrated that rapamycin enhances longevity in mice, even when initiated in relatively old animals (Harrison et al. 2009; Miller et al. 2011). Our laboratory was the first to describe mTOR activation as a feature of senescent human cells (Zhang et al. 2000), and mTOR activation has recently been described as a feature of aging tissues and cells (Nacarelli et al. 2015), suggesting that activation of the pathway is a component of age-related dysfunction in multiple settings and may underlie age-related functional decline. In addition, the mTOR pathway is a direct target of the IGF-1 signaling pathway, which is a major driver of aging (Kenyon 2010). Given the enormous volume of preclinical data supporting the use of rapamycin in age-related settings, defining clinical settings in which rapamycin may be safely used as an intervention and understanding the mechanisms by which rapamycin improves late-life functionality are critical issues in the field of biogerontology. Animal studies using companion dogs are ongoing (Urfer et al. 2017), and short-term studies have examined the safety and tolerability of rapamycin in older human subjects (Kraig et al. 2018). However, with the exception of a small feasibility study (Singh et al. 2016), the potential impact of rapamycin on the aging process in human tissue has not been evaluated. Thus, there is a critical need for clinical studies evaluating the impact of rapamycin on age-related functional decline. In terms of mechanism, senescence is a cell fate first described in culture as an intrinsic cellular aging process (Hayflick 1965). It is now clear that senescence can be triggered by stress, DNA damage, or metabolic dysfunction, and senescent cells contribute to age-related decline by influencing the microenvironment and through the production of inflammatory cytokines (He and Sharpless 2017; Tchkonia and Kirkland 2018). We and others have demonstrated that rapamycin can delay or prevent senescence in human cells (Bitto et al. 2010; Lerner et al. 2013). In the skin, senescence cells measured by increased expression of the p16^INK4A^ protein have been found to correlate with markers of aging such as elastic fiber morphology and wrinkling of the skin (Waaijer et al. 2016; Waaijer et al. 2012). Dermal atrophy, a significant problem in the elderly, can lead to skin fragility, impaired wound healing, and an increased rate of infections and complications following injury. Photoaging, manifested by fine wrinkling, dyspigmentation, and dull-appearing skin, is nearly ubiquitous in adults older than 50 years. In order to address the hypothesis that rapamycin treatment can ameliorate age-related disorder and dysfunction in human tissue through a reduction in senescence, we examined the impact of a topical formulation of rapamycin on markers of senescence and aging in human skin, using a placebo-controlled, exploratory trial design.

## Methods

### Patient recruitment and trial design

This prospective, randomized, placebo-controlled, exploratory study was conducted to test the hypothesis that a topical application of rapamycin (10 μM) would decrease the number of senescent cells in the skin and decrease markers of aging. Subjects were recruited at the Drexel University College of Medicine Department of Dermatology in Philadelphia, PA. The study protocol was approved by the Drexel University Institutional Review Board and was conducted in accordance with ethical principles outlined in the Declaration of Helsinki. Participants were greater than 40 years of age and had no history of diabetes or hypercholesterolemia. Full inclusion and exclusion criteria are included in the study protocol provided in Supplemental Materials. Participants enrolled in the study were provided with a container of rapamycin cream and a container of placebo (DMSO) in identical dispensers with labels for right or left hand and were instructed to apply the creams 0.5 cc (1 pump from the dispenser) to the dorsal side of each hand every 24 to 48 h in the evening before bed. Participants were evaluated at follow-up visits 2, 4, 6, and 8 months after initiation of the topical application. Wrinkles and clinical appearance of the skin were documented by photography at each clinic visit. Blood was drawn from consenting individuals (*n* = 13) at the 6-month visit to assess systemic delivery of rapamycin, and tissue from the dorsal surface of both hands was obtained by biopsy at final visit, using a 3-mm punch probe. Biopsies were divided into 2 sections for further analyses (histological evaluation, immunohistochemistry, isolation of total RNA). Rapamycin was measured in whole blood by an independent CLIA-certified laboratory (NMS Laboratories, Horsham, PA).

### Clinical assessment of dermal tissue

Clinical signs of aging were evaluated at each study visit. The appearance of the dorsal hands was evaluated using the Merz Hand Grading Scale, a validated measure where clinical hallmarks of aging such as prominent veins and tendons are associated with a higher score (Cohen et al. 2015). Fine wrinkles were assessed using an internal grading scale consisting of the following metrics: 0—absent, 1—slight, 2—evident, 3—marked, 4—very marked. Dyspigmentation and skin tone were evaluated using the Glogau Classification of Photoaging, modified to exclude assessment of facial wrinkling (Durai et al. 2012).

### Immunohistochemistry

Biopsies were fixed in 3% formalin for > 24 h, washed with PBS, and processed by the Pathology Diagnostics Laboratory in the Department of Pathology and Laboratory Medicine at Drexel University College of Medicine. Samples were processed and embedded in paraffin for sectioning. Immunohistochemistry was performed using the Ventana XT Ultra system (Roche Diagnostics) using the ultraView DAB detection kit. Antibodies and staining protocols were as follows: p16^INK4A^ (Enzo Life Sciences, ABS377-1000), p21^Cip1/Waf1^ (Cell Marque, DCS-60.2), tp53 (Ventana, D0-7), and cytokeratin 5/6 (Ventana, DS/16B4), with the exception of collagen VII which was a rabbit polyclonal antibody obtained from Abcam (ab93350). Antigen retrieval was performed using citrate buffer (36–64 min) except for collagen VII, which utilized enzymatic digestion (protease for 64 min). Blocking was performed for 24–32 min, and antibody incubation times ranged from 24 to 60 min. Antibodies and staining conditions are reported in Supplemental Table 1.

### RNA isolation and analysis

Total RNA was isolated from tissue biopsies using Qiagen RNA easy protocols. Quantity and integrity of RNA were initially evaluated using a NanoDrop 2000/2000C spectrophotomer (Thermo Fisher) and confirmed using fluometric analysis on a Qubit fluorimeter (Thermo Fisher). Finally, integrity was evaluated using a Bioanalyzer RNA 6000 Nano assay (Agilent). Expression analysis was performed using the nanoString nCounter expression system housed in Drexel University College of Medicine Department of Microbiology and Immunology. Gene expression analysis was performed using a custom gene expression panel that included collagen, keratin isoforms, inflammatory cytokines, and senescence-associated genes. Levels for two primary senescence-associated gene products, p16^INK4A^ and p21^Cip1/Waf1^, were below the level of detection in the tissue extract likely due to a low level of senescent cells relative to total tissue. Normalization was performed according to default settings in the nSolver software, and differential expression created using ratio versus rapamycin-treated is presented in Supplemental Table 2.

### Statistical analysis

Staining was evaluated as percent positive nuclei (p16INK4A, p21Cip1/Waf1, tp53) or staining intensity (collagen VII). Positive nuclei were scored using an automated cell scoring pipeline developed on the Aperio Systems. Collagen VII staining was scored based on independent visual assessment of immunohistochemical staining on a 1- to 4-point Likert scale. The data appear to follow a normal distribution; however, because of the small sample size, data were evaluated using both parametric and non-parametric testing.

## Results

Participants were recruited at the Drexel University College of Medicine Department of Dermatology. Demographics of the study participants are presented in Table 1. The primary endpoint for the study was the level of p16^INK4A^ protein based on the relationship of p16^INK4A^ to signs of aging in the skin (Waaijer et al. 2016; Waaijer et al. 2012). Secondary endpoints were additional markers of senescence, impact on skin appearance, and impact on histological markers of aging in the skin. Thirty-six participants were enrolled, of which 19 discontinued due primarily to a loss to follow-up (*n* = 9), followed by lack of compliance (*n* = 7), and drop out (*n* = 3) (Supplemental Table 2). No treatment-related adverse events were reported during the course of the study (adverse events reported included wrist fracture, leg fracture, shingles). Of the 17 participants who completed the study, 13 consented to a blood draw and skin biopsy, and 8 tissue samples produced reliable material for further analysis. No blood samples collected contained detectable levels of rapamycin as assessed by LC/MS/MS analysis (limit of detection, 1 ng/ml).

**Table 1:** Demographics of the study participants

Demographics	
Female	77%
Male	23%
White	*n* = 27
Black	*n* = 3
Hispanic	*n* = 3
Asian	*n* = 3
Total	*N* = 36
Detectable rapamycin > 1 ng/ml	
Blood draw	*n* = 13
	*n* = 0
Description	
Treatment-related adverse events	*n* = 0

**Fig. 1:**
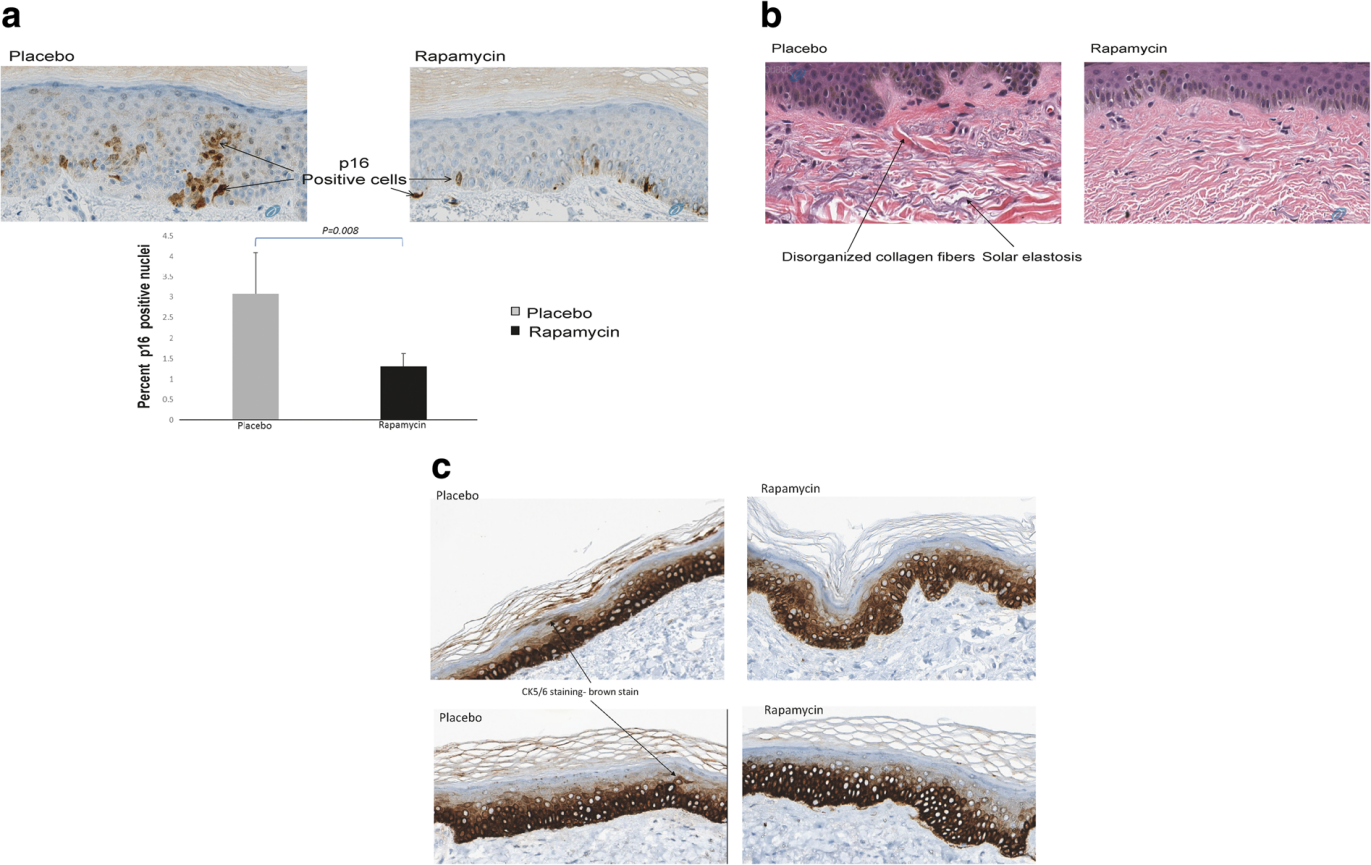
**a** Topical rapamycin treatment reduces expression of the senescence regulator p16^INK4A^. Human skin was treated with a formulation of 10 μM rapamycin or an identical formulation containing a vehicle control for 6–8 months; 0.5 cc of formulation was applied daily. Skin biopsies (*n* = 8) were taken at end of study and processed for immunohistochemistry as described in “Methods” section. Nuclear p16^INK4A^ was quantified using the Leica Aperio software system with a nuclear stain algorithm. **b** Topical rapamycin reduces signs of photoaging. Hematoxylin-eosin stain of human skin biopsies from the dorsal hands following application of topical cream containing rapamycin (10 μM) or placebo as in **a**. Histologic evidence of photoaging-actinic (solar) elastosis is indicated with an arrow. A reduction in the presence of these histologic markers of age-damaged skin was noted in multiple patient biopsies treated with rapamycin. **c** Topical rapamycin improves cytokeratin 5/6 distribution in human skin. Human skin biopsies from the dorsal hands of subjects following application of topical cream containing rapamycin or placebo as in **a** were stained with antibodies recognizing cytokeratin 5/6 (brown staining), a marker for basal cells in the epidermis. Note that staining in rapamycin-treated skin is more focally located in the basal layer of the skin while skin receiving placebo shows cytokeratin 5/6 staining in the stratum granulosum indicative of incomplete differentiation typical of aged skin

To evaluate the impact of rapamycin on cellular and molecular aspects of the skin, biopsies were analyzed for the expression of proteins related to senescence and aging. Immmunohistochemistry for p16^INK4A^ revealed a significant reduction (*P* = 0.008) in expression of the protein primarily in the epidermal layer of the skin (Fig. 1a). Levels of p21^Cip2^ and tp53 were also examined and showed a trend toward reduction in the rapamycin-treated samples, but the changes did not reach statistical significance. No evidence of pathologic changes to the skin, inflammation, or neutrophil infiltration was observed. Histologic evaluation of the tissue samples revealed a consistent reduction in the degree of solar elastosis in the rapamycin-treated skin samples (Fig. 1b). In addition, a more organized basal layer was apparent in the rapamycin-treated epidermis, and the expression of cytokeratin 5/6 was more tightly associated with the basal layer in the rapamycin-treated skin samples. Areas of cytokeratin 5/6 staining were noted in the stratum corneum in the placebo-treated samples (Fig. 1c), but were absent in the rapamycin-treated samples, although this result was not easily quantifiable and should be considered an observation. Such observations may be more amenable to study in preclinical models such as the companion dogs or mice in which larger sample sizes, and in vivo labeling approaches may prove in formative.

**Fig. 2:**
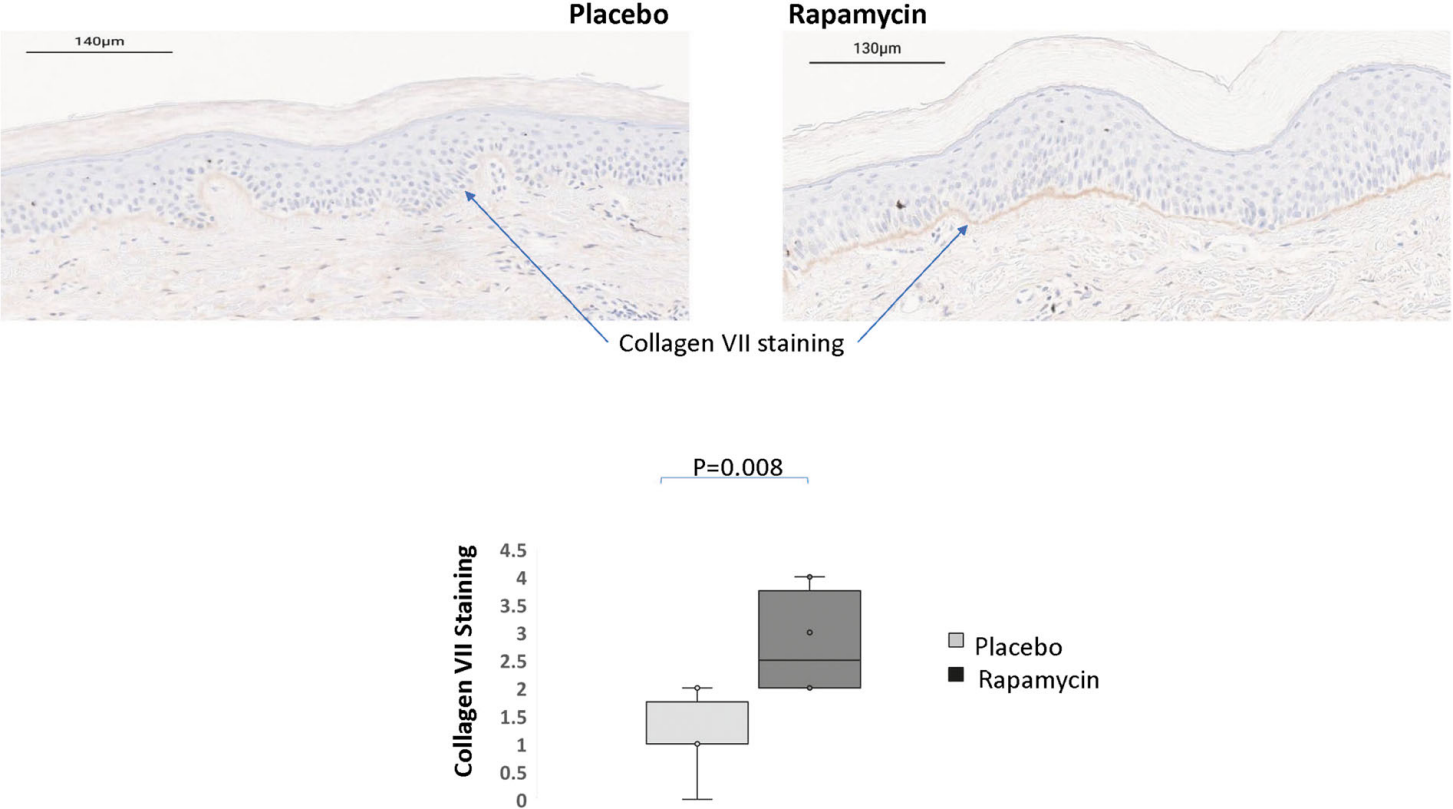
Topical rapamycin increases collagen VII in the basement membrane of human skin. Biopsies from patients (*n* = 6), placebo- and rapamycin-treated skin were processed for immunohistochemistry and stained using antibodies specific for the collagen VII protein

To further define the impact of rapamycin on the biology of the skin, an unbiased screen of 54 mRNAs relevant to senescence and skin biology was performed using a NanoString probe panel designed with gene products related to skin biology including keratin and collagen subtypes. Although the results were exploratory in nature due to the relatively small number of samples (*n* = 12), a reduction in the mRNA level for collagen VII was the most significant at *P* = 0.025 without multiple testing correction. The mRNA levels for tp53 were also reduced although not to a statistically significant level (Supplemental Table 3). Levels of p16^INK4A^ and p21^Cip1/Waf1^ were below the level of detection for NanoString, which was likely due to the low percentage of senescent cells relative to the total cell number in the tissue, combined with the fact that these mRNAs are of relatively low abundance within the cell. To further evaluate the impact of rapamycin treatment on collagen VII, immunohistochemistry was performed to assess protein levels. Several antibodies were evaluated as well as several approaches for antigen retrieval. A relatively strong antigen retrieval using protease treatment was required to visualize the classic basement membrane staining beneath the basal layer of epithelial cells. This analysis revealed that collagen VII protein was strongly increased in rapamycin-treated skin samples (Fig. 2). It appears that reduced mRNA levels reflect a feedback regulation induced by elevated protein levels.

**Fig. 3:**
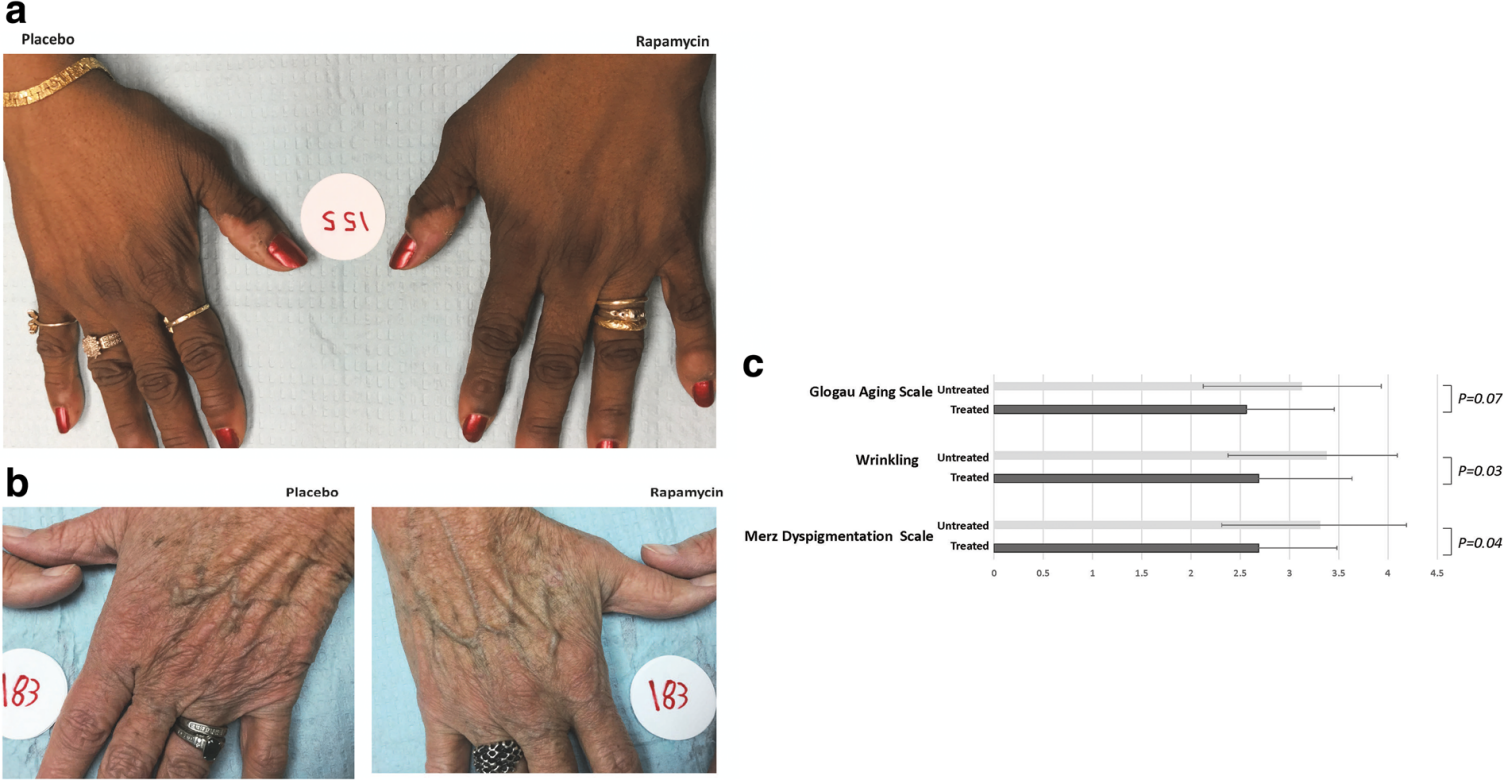
Rapamycin treatment improves clinical signs of aging in the skin. Clinical improvement in a 65-year-old woman (**a**) and a 67-year-old woman (**b**) following application of 10 μM rapamycin cream for 6–8 months. The placebo-treated hand is provided for comparison. in **c**, overall clinical improvement on 3 scales, the Glogau Classification of Photoaging, wrinkling measured on a 1-4 Likert scale, and the Merz Hand Grading Scale for aging skin, is presented for all subjects completing the study. Lower score on all scales correlates with reduced clinical severity

Clinical improvement of skin photoaging in participants receiving topical rapamycin was noted in the majority of subjects and included a decrease in fine wrinkles, an increase in dermal volume (Fig. 3a), a brighter and more even skin tone in treatment areas, and reduced sagging of the skin (Fig. 3b). Clinical assessment of the 13 subjects completing the study is presented in Table 2. Of the 13 subjects, all except 2 showed improvement in clinical signs of cutaneous aging. The most notable improvement was a decrease in the prominence of veins and tendons, a hallmark of age-related volume loss of the hands. Fine wrinkling was also improved, consistent with the increase in collagen VII. The dyspigmentation and dull appearance associated with photoaged skin was also consistently improved. These changes were evident approximately 4 months following the initiation of treatment, and continued improvement was noted upon subsequent visits.

**Table 2:** Clinical score for individual subjects at final visit

Subject number		Merz Grading Scale*	Wrinkling	Glogau Scale dyspigmentation
111	Treated	3	3	2
	Untreated	4	4	3
122	Treated	2	4	3
	Untreated	1	3	3
123	Treated	3	3	3
	Untreated	4	4	3
131	Treated	2	3	3
	Untreated	3	4	3
133	Treated	2	2	2
	Untreated	3	3	3
135	Treated	3	3	3
	Untreated	3	4	4
137	Treated	2	2	2
	Untreated	3	3	3
152	Treated	4	4	4
	Untreated	4	4	4
153	Treated	2	1	1
	Untreated	3	2	2
155	Treated	1	1	1
	Untreated	2	2	1
162	Treated	3	3	3
	Untreated	4	4	4
173	Treated	3	3	3
	Untreated	4	3	3
175	Treated	3	2	2
	Untreated	3	3	3
176	Treated	3	2	2
	Untreated	4	3	3
183	Treated	3	3	3
	Untreated	4	4	4
184	Treated	4	4	4
	Untreated	4	4	4

*Clinical rating scale where 0 = absent, 1 = slight, 2 = evident, 3 = marked, 4 = very marked. In all assessments, higher score is associated with more severe signs of aging

## Discussion

This study demonstrates a clear impact of rapamycin treatment on both the molecular signature associated with senescence and the clinical signs of aging in the skin. These data support the idea that a reduction in the burden of senescent cells underlies these improvements. The results could reflect a modification of the senescent cells present in the skin or a reduction in the number of senescent cells. Although rapamycin has been shown to reduce pro-inflammatory secretions produced by senescent cells (Laberge et al. 2015), the fact that p16^INK4A^ is reduced suggests that the absolute number of senescent cells in the epidermis is reduced. This implies that rather than simply modifying senescent cells present in the tissue, rapamycin treatment is either reducing the number of cells entering senescence or increasing the clearance of senescent cells. Based on our studies demonstrating that rapamycin prevents the senescence transition and improves functionality in vitro (Azar et al. 2018; Bitto et al. 2010; Lerner et al. 2013; Nacarelli et al. 2016), we favor the concept that rapamycin reduces entry into senescence, but we cannot rule out an additional role for clearance of senescent cells. Whether the reduction in senescent cells is due to reduced entry or increased clearance, a reduction in the burden of senescent cells would be expected to improve functionality. One notable limitation to our study is the use of a limited set of markers to evaluate senescent cells. Due to the limited sample obtained in the punch biopsies, we were unable to provide a more complete profile of senescence markers, and although p16^INK4A^ is strongly associated with senescence, it is not unique to the process. The use of in vitro or animals models is likely to provide a more thorough evaluation of the impact of rapamycin on senescence in multiple cell types.

Senescent cells produce pro-inflammatory cytokines, matrix metalloproteins, and reduced levels of anti-angiogenic factors, creating a secretory profile known as the Senescence-Associated Secretory Phenotype (SASP). The SASP creates an environment that is permissive to tumor formation and negatively impacts the stem cell niche. Although the SASP can be separated from p16^INK4A^ expression experimentally, in normal cells and tissues the two are tightly linked. Thus, we anticipate that rapamycin treatment reduces inflammatory cytokines in the skin, although the verification of this change represents a technical challenge due to the fact that such cytokines are present in picomolar amounts. One quantifiable aspect of skin biology that is improved by the rapamycin treatment is the incorporation of collagen VII into the basement membrane, which represents a functional measure of skin quality that is improved upon treatment with rapamycin. Collagen VII is essential for a functional skin barrier, and the levels of collagen VII decrease with age and specifically beneath wrinkles (Craven et al. 1997; El-Domyati et al. 2014). Although the mechanism whereby rapamycin may increase collagen VII protein levels is not clear at this time, the known effects of rapamycin on autophagy and intracellular trafficking of vesicles may allow for intracellular processing of misfolded collagen and increase proper localization at the cell periphery and basement membrane. Although the most prominent role for type VII collagen in disease is the presence of mutations associated with epidermolysis bullosa (Jarvikallio et al. 1997), the protein is widely distributed and functions as a critical element in anchoring fibrils (Sakai et al. 1986). Collagen VII has been reported to be present in astrocyte corpora amylacea in the eye (Wullink et al. 2015). Corpora amylacae are intracellular bodies found in astrocyte foot processes and axons which increase in abundance with age and appear to represent intracellular accumulations of insoluble proteins (Cavanagh 1999). These structures have recently been reported to contain fungal and microbial epitopes (Pisa et al. 2018), as well as both ubiquitin and p62/SQSTM1 (Auge et al. 2018), a ubiquitin-binding scaffold protein required for autophagic cargo loading (Bitto et al. 2014). These data link collagen VII with autophagic clearance of both misfolded proteins and extracellular pathogens, processes which may improve functionality in a variety of tissues. An additional possibility linking collagen VII with tissue function is cell-cell junctional integrity. Using more gentle antigen retrieval methods, we noted collagen VII staining in intracellular junctions which appeared increased in rapamycin treated skin. Although the data was not consistent enough for strong conclusions, it raises interesting possibilities for further investigation of the impact of rapamycin on epithelial or endothelial barrier functions. For example, rapamycin has been reported to improve blood brain barrier integrity in Alzheimer’s disease models (Van Skike et al. 2018), but the underlying mechanisms remain unclear, and improved cell-cell junctions or basement membrane integrity could enhance endothelial barrier function.

A notable aspect of this study is the use of such a low dose of rapamycin (10 μM, or 0.001%) for topical application. Topical treatment with higher concentrations (0.1–1%) has been employed for the treatment of tuberous sclerosis complex (TSC) in adults and children and has shown efficacy in the inhibition of the benign growths associated with the disorder without serious adverse events (reviewed in Darling, 2018) (Darling 2018). We chose to use rapamycin at a ten-fold lower dose because the concentrations used in TSC patients are intended to inhibit cell growth, while our aim was to improve cell function while maintaining proliferative potential and preventing senescence similar to our in vitro studies (Bitto et al. 2010; Lerner et al. 2013). This reduced dosing would be expected to pose even lower risk than the doses used for treatment of TSC. The positive impact of our treatment regimen suggests that age-related therapy with rapamycin may be feasible at doses far below those associated with side effects (Nguyen et al. 2019); however, this possibility will require careful evaluation in each specific clinical setting.

Beyond the impact on senescence, rapamycin is likely to alter cell function in all cells within the treated area. Although difficult to quantify directly, rapamycin will influence cellular function in all cell types due to the central nature of the mTOR complex in a variety of cellular processes (Johnson et al. 2015). Although no immune cell infiltrate was observed in any histological sections, we anticipate that rapamycin may impact local immune cell function. Langerhans cells identified by CD1a staining did not appear to differ between treated and untreated samples although functional differences may not be apparent based on simple numbers and processes.

In summary, we present the first evidence that rapamycin treatment improves function and reduces markers of aging in human tissue. The results support further investigation into clinical settings in which rapamycin treatment might provide similar benefits in older individuals.

## Electronic supplementary material

ESM 1 (DOCX 28 kb)
