# Supplementary material for: Topical rapamycin reduces markers of senescence and aging in human skin: an exploratory, prospective, randomized trial
Source: GeroScience. 2019 Nov 25;41(6):861–9. doi: 10.1007/s11357-019-00113-y (PMC6925069; doi:10.1007/s11357-019-00113-y)
Supplement: Supplementary file 1 — (DOCX 28 kb) [file 11357_2019_113_MOESM1_ESM.docx]

**Supplemental Table 1: Antibodies and Immunohistochemistry Conditions**

| **Antigen** | **Clone** | **Species/Type** | **Dilution** | **Antibody Vendor** | **Antigen Retrieval** | **Blocking** | **Amplification** | **Detection  Kit** |
| --- | --- | --- | --- | --- | --- | --- | --- | --- |
| Collagen VII | Polyclonal | Rabbit Polyclonal | 1 to 100 | Abcam | Protease 1 | None | None | Ultraview Universal DAB |
| Cytokeratin 5/6 | D5/16B4 | Mouse Monoclonal | Predilute | Ventana | Ultra CC1 | None | None | Ultraview Universal DAB |
| p16 | INK4a | Mouse Monoclonal | 1:50 | Enzo Life Sciences | Ultra CC1 72 min | | Optiview Amp Kit | Optiview DAB |
| p21 | DCS-60.2 | Mouse Monoclonal | Predilute | Cell Marque | Ultra CC1 64 min | | None | Ultraview |
| p53 | DO-7 | Mouse Monoclonal | Predilute | Ventana | Ultra CC1 36 min | | None | Universal DAB |

**Supplemental Table 2: Participants Discontinued or Lost to Follow-up**

|  | **0 to 2  Months** | **2 to 4  Months** | **4 to 6  Months** | **6 to 8 Study Months End** |  |
| --- | --- | --- | --- | --- | --- |
| **Drop Out**  **No. (% Total)**  **Active**  **No. (% Total)** | **10 (27.8)**    **26 (72.2)** | **4 (11.1)**  **22 (61.1)** | **4 (11.1)**  **18 (0.5)** | **1 (2.8) 19 (52.8)**  **17 (47.2) 17 (47.2)** |  |
| **Discontinued**  **No.** | **4** | **2** | **3** | **1 10** |  |
| **Lost to Follow-up No.** | **6** | **2** | **1** | **0 9** |  |

Subject 121 was lost to follow up in June.

Subject 138 dropped out due to non-compliance.

Subject 139 dropped out due to non-compliance because of vacation and a shingles infection on the fingers.

Subject 144 dropped out, no longer wished to participate.

Subject 158 dropped out due to non-compliance and difficulty in making follow up appointments.

Subject 132 dropped out due to non-compliance.

Subject 142 dropped out due to non-compliance. The subject was unable to keep up with the routine.

Subject 154 no longer wished to participate.

Subject 174 dropped out due to a broken wrist and was unable to use the creams.

**Other**

Subject 171 had an injury to leg and knee and was unable to make follow up appointments. The subject is still interested in the study and will contact the research team when the injury has healed.

Subject 191 has not started using the creams yet. She started traveling soon after being enrolled.

**Supplemental Table 3. Differential Expression Analysis Based on NanoString RNA Count Results**

| Probe Name | Accession # | NO vs RAPA | DE Call  NO vs RAPA | | | *P*-value  NO vs RAPA | | Lower 95% CI  NO vs RAPA | | | Upper 95% CI NO vs RAPA | |
| --- | --- | --- | --- | --- | --- | --- | --- | --- | --- | --- | --- | --- |
| Col12A1 | NM_004370.5 | 0.79 | N/A | | 0.509435 | | 0.35 | | | | | 1.75 |
| Col7A1* | NM_000094.2 | 1.98 | N/A | | 0.025027 | | 1.1 | | 3.57 | | | |
| ELN | NM_001081754.1 | 1.5 | N/A | | 0.377317 | | 0.54 | | 4.31 | | | |
| FBLN5 | NM_006329.3 | 1.2 | N/A | | 0.424635 | | 0.69 | | 2.1 | | | |
| FBN1 | NM_000138.3 | 1 | N/A | | 0.987229 | | 0.53 | | 1.85 | | | |
| GBL | NM_001199173.1 | 1.53 | N/A | | 0.26549 | | 0.66 | | 3.56 | | | |
| HPSE | NM_006665.3 | 1.33 | N/A | | 0.407744 | | 0.61 | | 2.89 | | | |
| HSPG2 | NM_005529.5 | 0.87 | N/A | | 0.695815 | | 0.39 | | 1.95 | | | |
| IL18 | NM_001562.2 | 1.38 | N/A | | 0.247373 | | 0.76 | | 2.49 | | | |
| IL1a | NM_000575.3 | 1.48 | N/A | | 0.510111 | | 0.37 | | 5.9 | | | |
| IL1b | NM_000576.2 | 1.8 | N/A | | 0.448578 | | 0.3 | | 10.73 | | | |
| IL6 | NM_000600.3 | 1.98 | N/A | | 0.331693 | | 0.42 | | 9.34 | | | |
| IL8 | NM_000584.2 | 1.44 | N/A | | 0.652137 | | 0.24 | | 8.78 | | | |
| IVL | NM_005547.2 | 1.08 | N/A | | 0.787317 | | 0.58 | | 2 | | | |
| KRT1 | NM_006121.2 | 0.72 | N/A | | 0.698648 | | 0.11 | | 4.91 | | | |
| KRT10 | NM_000421.3 | 1.09 | N/A | | 0.75914 | | 0.58 | | 2.05 | | | |
| KRT14 | NM_000526.4 | 1.44 | N/A | 0.179527 | | | 0.81 | | 2.53 | | | |
| KRT16 | NM_005557.3 | 1.12 | N/A | 0.821894 | | | 0.37 | | 3.35 | | | |
| KRT5 | NM_000424.2 | 2.17 | N/A | 0.127705 | | | 0.76 | | 6.16 | | | |
| LAMA5 | NM_005560.3 | 2.47 | N/A | 0.120979 | | | 0.73 | | 8.37 | | | |
| LAMB2 | NM_002292.3 | 0.97 | N/A | 0.879165 | | | 0.63 | | 1.51 | | | |
| LaminB1 | NM_005573.2 | 1.21 | N/A | 0.516956 | | | 0.63 | | 2.33 | | | |
| MET | NM_001127500.1 | 1.47 | N/A | 0.139089 | | | 0.86 | | 2.51 | | | |
| MMP1 | NM_002421.3 | 1.79 | N/A | 0.410381 | | | 0.36 | | 8.99 | | | |
| MMP2 | NM_004530.2 | 0.7 | N/A | 0.27596 | | | 0.33 | | 1.49 | | | |
| MMP3 | NM_002422.3 | 1.19 | N/A | 0.830865 | | | 0.18 | | 7.92 | | | |
| Mtor | NM_004958.3 | 1.27 | N/A | 0.386336 | | | 0.7 | | 2.29 | | | |
| NFKB1 | NM_003998.2 | 1.49 | N/A | 0.235671 | | | 0.71 | | 3.13 | | | |
| PBRM1 | NM_018313.4 | 1.46 | N/A | 0.329793 | | | 0.63 | | 3.35 | | | |
| PTEN | NM_000314.3 | 1.02 | N/A | 0.92615 | | | 0.68 | | 1.53 | | | |
| SETD2 | NM_014159.6 | 1.02 | N/A | 0.848804 | | | 0.76 | | 1.39 | | | |
| SIRT1 | NM_012238.4 | 1.22 | N/A | 0.239769 | | | 0.85 | | 1.74 | | | |
| SIRT2 | NM_012237.3 | 1.25 | N/A | 0.064054 | | | 0.98 | | 1.61 | | | |
| Serpine1 | NM_000602.2 | 1.23 | N/A | 0.758343 | | | 0.28 | | 5.43 | | | |
| TP53 | NM_000546.2 | 1.63 | N/A | 0.052227 | | | 0.99 | | 2.68 | | | |
| TSP-1 | NM_003246.2 | 0.89 | N/A | 0.653018 | | | 0.49 | | 1.61 | | | |
| VEGFA | NM_001025366.1 | 1.78 | N/A | 0.103378 | | | 0.86 | | 3.65 | | | |
| VHL | NM_000551.2 | 1.19 | N/A | 0.115875 | | | 0.95 | | 1.51 | | | |
| col1A1 | NM_000088.3 | 1.21 | N/A | 0.779451 | | | 0.25 | | 5.95 | | | |
| col1A2 | NM_000089.3 | 1.14 | N/A | 0.709534 | | | 0.52 | | | 2.47 | | |
| col2A1 | NM_001844.4 | 1.88 | N/A | 0.505421 | | | 0.21 | | | 17 | | |
| col3A1 | NM_000090.3 | 0.61 | N/A | 0.466473 | | | 0.14 | | | 2.72 | | |
| col4A1 | NM_001845.4 | 1.48 | N/A | 0.384284 | | | 0.55 | | | 3.99 | | |
| col4A2 | NM_001846.2 | 1.43 | N/A | 0.308571 | | | 0.67 | | | 3.02 | | |
| col4A3 | NM_000091.4 | 2.27 | N/A | 0.237442 | | | 0.46 | | | 11.22 | | |
| col4A4 | NM_000092.4 | 1.67 | N/A | 0.45312 | | | 0.34 | | | 8.14 | | |
| col5A1 | NM_000093.3 | 1.47 | N/A | 0.076359 | | | 0.95 | | | 2.27 | | |
| macroH2A | NM_001040158.1 | 1.31 | N/A | 0.065245 | | | 0.98 | | | 1.76 | | |
| p16 | NM_000077.4 | 1.41 | N/A | 0.700947 | | | 0.16 | | | 12.72 | | |
| p21 | NM_000389.4 | 1.36 | N/A | 0.20219 | | | 0.81 | | | 2.29 | | |
| Actin | NM_001101.2 | 1.22 | N/A | 0.245693 | | | 0.84 | | | 1.78 | | |
| POLR1B | NM_019014.3 | 0.97 | N/A | 0.917703 | | | 0.42 | | | 2.2 | | |
| POLR2A | NM_000937.2 | 1.12 | N/A | 0.608661 | | | 0.69 | | | 1.82 | | |
| Tubullin | NM_178014.2 | 0.76 | N/A | 0.281376 | | | 0.43 | | | 1.33 | | |

*Denotes collagen VII mRNA
